# How experience modulates semantic memory for food: evidence from elderly adults and centenarians

**DOI:** 10.1038/s41598-018-24776-3

**Published:** 2018-04-24

**Authors:** Miriam Vignando, Marilena Aiello, Francesco Foroni, Gabriella Marcon, Mauro Tettamanti, Raffaella I. Rumiati

**Affiliations:** 1 Neuroscience and Society Laboratory, SISSA, Trieste, Italy; 2 School of Psychology, Faculty of Health Sciences, Australian Catholic University, Sydney, Australia; 3 DAME, University of Udine, Udine, Italy; 4 Department of Medical, Surgical and Health Sciences, University of Trieste, Trieste, Italy; 5 Laboratory of Geriatric Neuropsychiatry, IRCSS - Istituto di Ricerche Farmacologiche Mario Negri, Milan, Italy; 6 ANVUR, Rome, Italy

## Abstract

In order to make sense of the objects we encounter in everyday life we largely rely on previous knowledge stored in our semantic memory. Semantic memory is considered dependent on lifelong experience and cultural knowledge. So far, a few studies have investigated the role of expertise on the organization of semantic memory, whereas life-long experience has largely been overlooked. In this study, we investigated this issue using food concepts. In particular, we administered different semantic tasks using food (natural and transformed) and non-food (living and non-living things) as stimuli to participants belonging to three different age cohorts (56–74, 75–91, 100–108), who were also asked to report on the dietary habits held throughout their life. In addition, we investigated to what extent psycholinguistic variables influence the semantic performance of different age cohorts. Results showed that Centenarians recognized natural food better than transformed food, while the other two groups showed the opposite pattern. According to our analyses, experience is responsible for this effect in Centenarians, as their dietary habits seem to suggest. Moreover, significant correlations between picture naming and age of acquisition, familiarity and frequency were observed. This study indicates that lifelong experience can shape conceptual knowledge of food concepts, and that semantic memory is less resilient to aging than initially thought.

## Introduction

Semantic memory stores conceptual knowledge that subserves several tasks including recognition of objects and actions, as well as the production and understanding of sentences. Unlike fluid intelligence, considered independent of learning, experience, and education^1^, semantic memory is part of ‘crystallized intelligence,’ and is dependent on lifelong experience^2,3^ and cultural knowledge^4,5^. This implies that we can recognize and access meaningful information about categories of objects that we have encountered before in our lives.

In this view, semantic knowledge can be argued to be shaped by experience. The studies that have tested this hypothesis have to date been limited to a specific instance of experience, that is *expertise*, defined as an exceptional skill or performance in a given domain that requires extensive training (for a review, see Hoffman^6^). Expertise has also been found to entail specific patterns of neural activity, such as visual tuning, attention and memory^7–9^ (for a review, see Harel, Kravitz and Baker^10^). Importantly, training may also be achieved in a limited amount of time, as apparent in the study by Gauthier and Tarr^11^ in which participants were trained to become experts on an invented category of objects, the *Greebles*. Compared to novices, experts’ intensive training improved both accuracy and reaction times in a recognition task. Specifically, studies on expertise have investigated differences in semantic memory of experts - truly exceptional people at a specific domain of knowledge^12^ (e.g., ornithologists^13–15^, dog show judges^16^, car experts^17^, chess experts^8^) - by comparing their performance to that of novices or even naïve participants - defined as *‘totally ignorant of a domain*’ in Hoffman’s taxonomy^6^. Expertise has been consistently found to enhance the ability to list features of exemplars at the subordinate level (e.g., ‘robin’ rather than ‘bird’), to categorize and name exemplars of one’s domain of expertise (e.g.^18^), and to recognize complex^19^ or novel objects (e.g. classes of objects created with algorithms^11,20^).

However, individuals may possess considerable experience in a given domain and yet they might not be considered experts^21^. Their *experience* corresponds to the knowledge they acquired over a number of years and, as such, it depends on the degree or frequency with which they have been exposed to a stimulus, rather than on the amount of training or practice.

As part of experience, cultural knowledge is likely to play a role as well in shaping semantic memory, as revealed by ethno-scientific studies. For instance, Dougherty^22^ observed that the Tzeltal population’s recognition of plants and animals occurs at a more specific level (*folk genera*, e.g., oak) rather than at the superordinate level (e.g. *tree*). In addition, it has been shown that memory and similarity judgments were influenced by cross-cultural factors in two different tribes^23^.

Moreover, as different age-cohorts are likely to be exposed to different cultural and socio-economical conditions, some types of knowledge change across generations^24–26^. In Poon and Fozard’s^27^ study, young and elderly adults were presented with objects that, compared to commonly familiar stimuli, were either obsolete and unfamiliar to young adults but familiar for elderly adults or, *vice versa*, too modern or unfamiliar to elderly adults but familiar to young adults. These authors found that naming of either group was influenced by the degree of familiarity with the object categories. Likewise, Bäckman and Karlson^28^ investigated the effect of datedness of information in two different age groups, by asking young adults and old adults questions about events that took place in the period from 1930 to 1950, and from 1970 to 1983. Similarly to Poon and Fozard’s findings, they observed that elderly adults performed better on dated information and young adults on contemporary information. Taken together these results suggest that investigating semantic memory in different age-cohorts may be a good model for exploring the relationship between semantic knowledge and experience.

In the present study, we assessed the role of experience in food recognition as established in life-long eating habits. To this end we administered different semantic tasks using food and non-food stimuli to participants of three different age cohorts (Young Old, aged 56–74, Old Old, aged 75–91, and Centenarians aged 100–108), and asked them to report on the dietary habits held throughout their life. We hypothesized that the participants of the different age-cohorts went through different eating experiences and cultural contexts which, in turn, differentially shaped their semantic memory about food. In addition, the enrollment of the different age cohorts was expected to inform us on how semantic memory declines throughout aging. Further predictions were made with respect to the type of task and the type of stimuli used. On one hand, based on the combined evidence on naming abilities and ageing, particularly participants’ naming was expected to decline (see e.g.^29–33^). As to the stimuli, we expected food representations to be more resilient to detrimental processes, as recently demonstrated^34^. Furthermore the items we employed could denote either natural foods, such as an apple or a tomato, or transformed food, that is food that underwent an organoleptic transformation, such as pizza or hamburger. The full design of all tasks included also non-food items distinguished in natural (living things) and transformed (nonliving things). The distinction between natural and transformed food has been included as previous studies suggested that these two types of concepts might be differentially represented in semantic memory^34–36^, in analogy with what proposed for living and non-living things, respectively^2^.

## Results

The group of Young Old adults and the group of Old Old adults were matched for education [*t* (41) = −0.66, *p* = 0.51] and MMSE (*t* (41) = −0.12, *p* = 0.26), while Centenarians’ MMSE scores were significantly lower compared to both Young Old [*t* (40) = −4.12, *p* < 0.001] and Old Old [*t* (25.5) = −4.25, *p* < 0.001] adults. Moreover, their education level resulted marginally, but not significantly, lower than those of the Young Old adults [*t* (40) = −1.70, *p* = 0.10] and of the Old Old adults [*t* (40) = −1.82, *p* = 0.07]. See Table 1.

**Table 1:** Mean age, education and MMSE of the three age groups.

	**Young Old Adults**	**Old Old Adults**	**Centenarians**
	N = 24 (15 F)	N = 19 (13 F)	N = 18 (12 F)
*Age, Education and MMSE Meand and Range Across the three Age groups*			
**Age**	67.75 (±6.69)	79.15 (±1.00)	102.38 (±2.33)
range	(51–74)	(75–91)	(100–108)
**Education**	8.79 (±2.35)	9.52 (±4.81)	7.22 (±3.20)
range	(5–13)	(5–18)	(2–13)
**MMSE**	28.27 (±1.87)	27.47 (±2.01)	23.20 (±4.33)
range corrected	(24–30)	(24–30)	
range raw	(26–30)	(24–30)	(12–30)
	ns	ns	ps < 0.001

### Effect of Group on lexical-semantic processing

#### Naming

We observed a significant main effect of *Group* [*F* (2, 56) = 50.95, MSE = 721.22*, p* < 0.001, η^2^ = 0.65], with Centenarians being outperformed by both Young Old [*p* < 0.001, CI (−16.91, −10.14])] and Old Old adults [*p* < 0.001, CI (−16.38, −8.92)], with Old Old and Young Old adults not differing between each other [*p* = 1, CI (−1.99, 3.74)], and a significant interaction *Group x Stimulus type xlevel of artificiality* (that is, natural versus artificial items) [*F* (2, 56) = 38.04, MSE = 19.02, *p* < 0.001, η^2^ = 0.24]. No other effects emerged and, in particular, food and non-food items were named with the same accuracy by all three groups. To further investigate the interaction *Group x Type of item x level of artificiality*, we run two MANCOVAs *group* x *level of artificiality*, separately for the *food* and *non-food* items.

#### *Naming food items* (natural, NF and transformed, TF)

**Figure 1:**
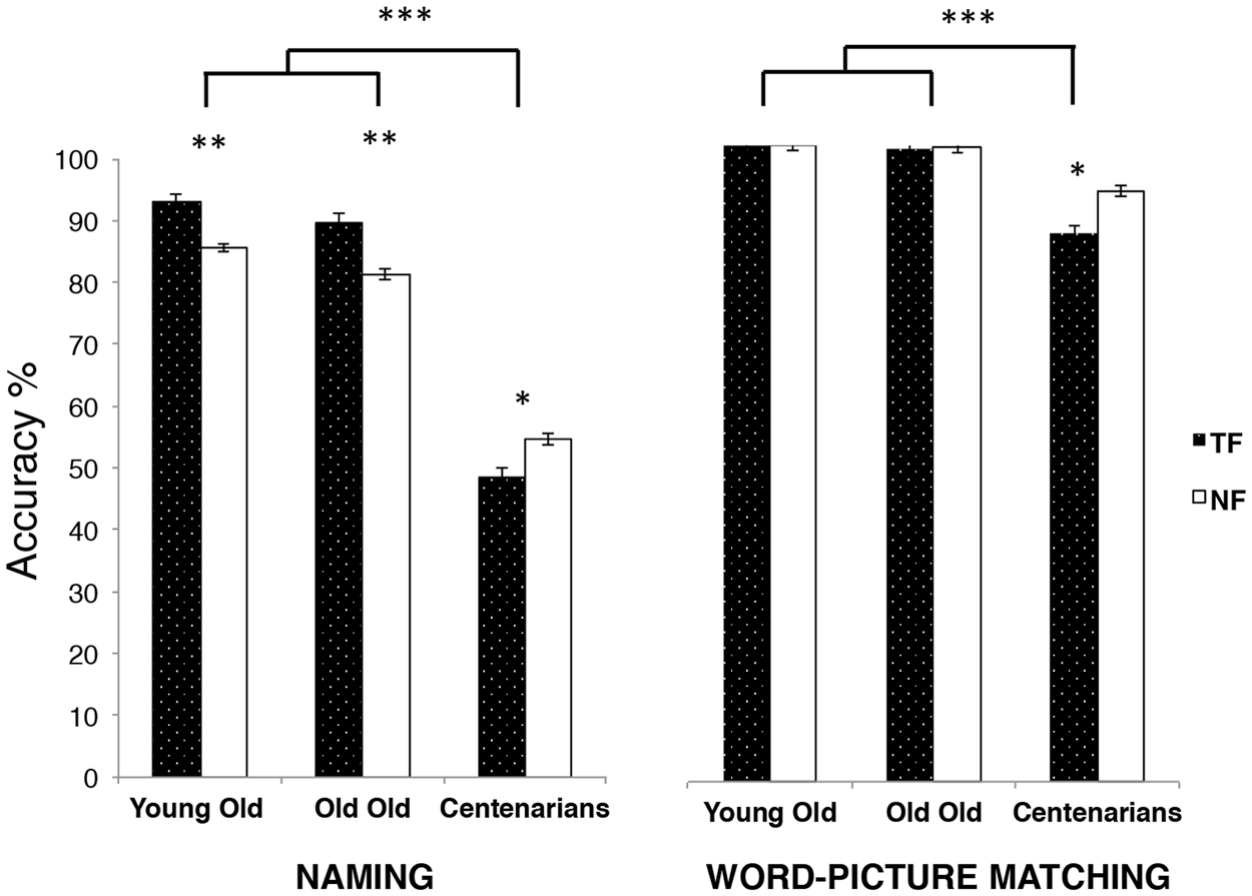
Mean accuracy for natural vs. transformed food across the three groups and for two the tasks. In the naming task, besides the group effect, with Centenarians performing significantly worse than Young Old and Old Old adults, there is a significant Type of food x Group interaction, with Young Old and Old Old adults showing and advantage for transformed food and Centenarians showing the opposite pattern. In the Word-Picture matching task we observe a similar patter, with Centenarians performing significantly worse than both Young Old and Old Old and showing a significantly better performance at natural versus transformed food, whereas the other two groups did not show any difference (***p < 0.001, **p < 0.01, *p < 0.05).

The analysis showed a main effect of Group [*F* (2, 56) = 55.5, MSE = 407.09, *p* < 0.001, η2 = 0.55] as well as a significant *Group x level of artificiality* interaction [*F* (2, 56) = 8.36, MSE = 12.68*, p* < 0.01, η^2^ = 0.23]. Transformed foods were named better than natural foods by Young Old adults [M_NF_ = 15.41 ± 0.25; M_TF_ = 16.75 ± 0.37; *p* < 0.01, 95% CI (0.62, 2.3)] and Old Old adults [M_NF_ = 14.63 ± 0.37; M_TF_ = 16.15 ± 0.45; *p* < 0.01, 95% CI (0.57, 2)] whereas centenarians named natural food (M_NF_ = 9.83 ± 0.78) significantly better than the transformed one [M_TF_ = 8.61 ± 0.69, *p* = 0.02, 95% CI (0.15, 2.1)] (see Fig. 1). Please note that the difference in Young Old adults and Old Old adults were not significant when a generalized linear model with binomial family was run (see Supplementary Information S9).

#### *Naming non-food items* (living and non-living)

The MANCOVA revealed, beyond the main effect of Group [F (2, 56) = 32.27, MSE = 316.95, *p* < 0.001, η2 = 0.54], a main effect of *level of artificiality* [F (1, 56) = 56.0, MSE = 9.66, *p* = 0.05, η2 = 0.07], with non-living things being overall named more accurately than living things. A significant *Education level x non-food type* interaction was also observed, possibly due to the fact that some of the non-food items used were more difficult than others for our participants (see Supplementary Information S1 for means and standard deviations).

#### Word-Picture matching

The Mann-Whitney test confirmed that Centenarians performed significantly worse than Young Old (food, *U* = 24, *p* < 0.001, non-food, *U* = 25.5, *p* < 0.001) and Old Old adults (food, *U* = 23.5, *p* < 0.001, non-food, *U* = −25, *p* < 0.001), whereas these two groups did not differ from each other (food, *U* = 204 *p* = 0.1, non-food, *U* = 189.5, *p* = 0.09). Moreover, no difference was observed between food and non-food in Old Old adults (*Z* = −0.58, *p* = 0.57), in Centenarians (*Z* = −0.68, *p* = 0.5), and in Young Old adults, who perform at ceiling (M_TF_ = 36 ± 0, M_NF_ = 36 ± 0).

Concerning food items, Centenarians performed significantly worse in both natural and transformed food recognition with respect to Young Old adults (NF: *U* = 48, *p* < 0.001; TF: *U* = 36, *p* < 0.001) and Old Old adults (NF: *U* = 44, *p* = *p* < 0.001; TF: *U* = 35.5, *p* < 0.001), whereas these two groups did not differ from each other (*U* = 216, *p* = 0.26, for both categories). Wilcoxon rank sum tests revealed that Young Old performed at ceiling at both natural and transformed food (M_NF_ = 18 ± 0; M_TF_ = 18 ± 0) and that Old Old adults did not show any difference in performance (M_NF_ = 17.94 ± 0.05; M_TF_ = 17.89 ± 0.11; *Z* = −0.45, *p* = 0.66). By contrast, Centenarians recognized natural food significantly better than transformed food (M_NF_ = 16.72 ± 0.28; M_TF_ = 15.5 ± 0.47; *Z* = −2.62, *p* = 0.01). This analysis was repated with a generalized linear model with binomial family, confirming the centenarians’ greater impairment in recognizing transformed food. This analysis also showed a difference in Old Old adults’ performance, with natural food being better recognized than transformed food (see Supplementary Information S9). However, as shown by the mean values, Old Old adults performed at ceiling with both categories.

For what concerns non-food items, beyond the worse group performance of Centenarians, no significant differences were observed (see Supplementary Information S2 for details and S1 for means).

#### Categorization task

Since all of the three groups showed a ceiling effect, results concerning the categorization task are reported in the Supplementary Information (S8).

#### Error Analysis for the naming task

Consistently with the accuracy results, the error analysis using the Wilcoxon test revealed that Centenarians made significantly more semantic errors for transformed food than for natural food (M_NF_ = 2.44 ± 1.38, M_TF_ = 4.39 ± 2.25, *Z* = −2.60, *p* = 0.01), whereas the Young Old adults (M_NF_ = 1.42 ± 1.26, M_TF_ = 0.68 ± 0.50, *Z* = −2.121, *p* = 0.03) and Old Old adults (M_NF_ = 0.45 ± 0.72, M_TF_ = 0.20 ± 0.50, *Z* = −1.987, *p* = 0.05) made more semantic errors for natural food (for details, see Supplementary Information, S3 and S4).

### The role of psycholinguistic variables in the three different age cohorts

#### Naming food items

Correlational analyses between psycholinguistic variables and food naming in the three groups, can be found in Table S5 of the supplementary material. These analyses showed no significant correlations between naming performance and any of the psycholinguistic variables for the Young Old adults, whereas a positive correlation was observed for familiarity in the Old Old adults; Centenarians’ food naming performance positively correlated with familiarity and frequency and negatively with age of acquisition, suggesting that psycholinguistic variables may have a differential predictive value regarding the degradation of semantic memory. Specifically, familiarity seems to strongly influence the performance after 75 years of age, whereas after 100 years also frequency of use and age of acquisition have an effect. Regression analyses were performed on the food naming accuracy on the 36 food items for each of the age groups, using familiarity, frequency, number of letters and age of acquisition as predictors (see S6). The model resulted significant for Centenarians only [*F* (4, 30) = 3.66, with *p* = 0.01 and *r*^2^ = 0.33], with none of the single variable adding statistical significance.

#### Naming of natural and transformed foods

In Centenarians, these analyses showed a significant positive correlation between naming natural food and familiarity (*r* = 0.68, *p* < 0.01), and a significant but negative correlation between naming natural food and age of acquisition (*r* = −0.55, *p* = 0.02). None of these correlations resulted significant in the other two groups.

Caloric content negatively correlated with naming natural food in Old Old, but not in Young Old adults or Centenarians, while it positively correlated with transformed food in the Young Old adults and the Old Old adults but not in Centenarians (see Table 2). Regression analyses were performed on both natural and transformed food naming performance using only age of acquisition and familiarity as predictors, since none of the other psycholinguistic variables significantly correlated with the performance (see Supplementary Table S7). The model resulted significant for Centenarians only [*F* (2, 17) = 7.3, *r*^2^ = 0.49, *p* = 0.01] and concerning just natural food, with only familiarity adding statistical significance (*β* = 43.75, *p* = 0.03).

**Table 2:** Pearson correlations between psycholinguistic variables, calorie content and natural and transformed food naming scores for the three age groups.

	Written Frequency	Letters	AofA	Familiarity	Calorie Content
***Correlation Analyses between Natural Food Naming Accuracy, Psycholinguistic Variables and Calorie Content***					
Young Old adults	*r* = 0.30	*r* = −0.25	*r* = 0.12	*r* = −0.06	*r* = −0.19
	*p* = 0.24	*p* = 0.32	*p* = 0.63	*p* = 0.82	*p* = 0.44
Old Old adults	*r* = 0.35	*r* = −0.13	*r* = −0.15	*r* = 0.35	*r* = −0.47
	*p* = 0.18	*p* = 0.60	*p* = 0.57	*p* = 0.16	*p* = 0.05
Centenarians	*r* = 0.43	*r* = −0.02	*r* = −0.55**	*r* = 0.68**	*r* = −0.37
	*p* = 0.08	*p* = 0.92	*p* = 0.03	*p* < 0.01	*p* = 0.14
***Correlation Analyses between Transformed Food Naming Accuracy, Psycholinguistic Variables and Calorie Content***					
Young Old adults	*r* = 0.00	*r* = −0.01	*r* = −0.20	*r* = 0.29	*r* = 0.49
	*p* = 0.99	*p* = 0.97	*p* = 0.43	*p* = 0.25	*p* = 0.04
Old Old adults	*r* = 0.11	*r* = 0.00	*r* = −0.35	*r* = 0.35	*r* = 0.52*
	*p* = 0.67	*p* = 0.10	*p* = 0.15	*p* = 0.15	*p* = 0.03
Centenarians	*r* = 0.23	*r* = 0.33	*r* = −0.43	*r* = 0.29	*r* = 0.27
	*p* = 0.23	*p* = 0.18	*p* = 0.08	*p* = 0.24	*p* = 0.28

*Correlation is significant at the 0.05 level (2-tailed).

**Correlation is significant at the 0.01 level (2-tailed).

#### Dietary Habits

**Figure 2:**
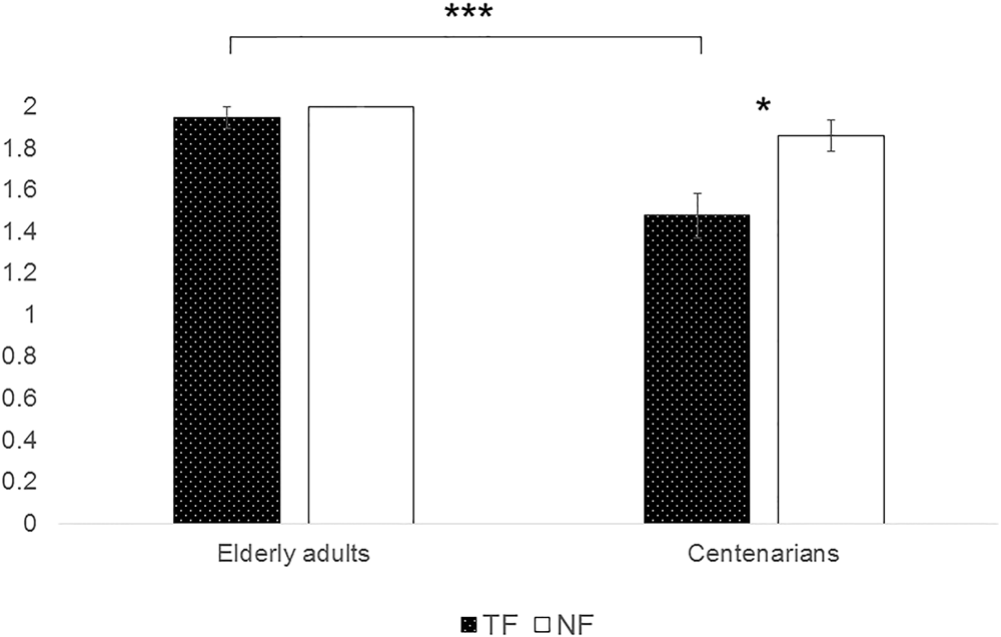
Dietary habits for Centenarians and Elderly Adults. Wilcoxon tests revealed that, compared to younger elderly adults, Centenarians have eaten significantly less frequently transformed foods than natural foods (*p < 0.05, ***p < 0.001).

The life dietary habits were collected for a subset of participants (N = 37) including Centenarians (N = 18) and 19 participants from the two groups of elderly adults (M_age_ = 78.52 ± 4.66, range 70–91). Participants were asked to rate on a scale ranging from ‘Often’ (scored as 2), to ‘Seldom’ (scored as 1), to ‘Never’ (scored as 0), the extent to which they had eaten a particular type of food in their lifetime. We aggregated the transformed foods (pasta, processed meats, sweets) and the natural foods (raw fruit and raw vegetables), and performed the Wilcoxon signed ranks test. Centenarians (N = 18) confirmed to have eaten natural food significantly more frequently than transformed food (M_TF_ = 1.48 ± 0.10; M_NF_ = 1.86 ± 0.07, z = −2.73, *p* = 0.04), whereas the subgroup of elderly adults showed no differences (M_TF_ = 1.95 ± 0.05; M_NF_ = 2 ± 0.00) (see Fig. 2). Moreover, we performed a Mann-Whitney test comparing the frequency of consumption of both natural and transformed food in the two groups, which revealed that the subgroup of elderly adults ate transformed food more frequently with respect to centenarians (*U* = 41.50, *p* < 0.001).

To test whether the frequency of consumption effectively predicted Centenarians’ naming accuracy, a bivariate linear regression with a ‘frequency index’ [freq_NF_–freq_TF_] – that is a difference in the frequency of consumption between natural and transformed food – was used as independent variable and an ‘accuracy index’ [naming accuracy _NF_–naming accuracy _TF_] the naming accuracy on the two categories, that is the difference in naming transformed and natural food. The regression model resulted significant [*F* (1, 35) = 4.98, *p* = 0.03, *r*^2^ = 0.13], with frequency of consumption effectively predicting naming performance. Moreover, the bootstrap method (100000 iterations) was applied to estimate the confidence interval for the regression parameter, which resulted significantly different from 0 (*p* = 0. 02, CI [0.02, 0.18]).

## Discussion

The main findings of the present study are the following. Food recognition degrades differently depending on the age of the participants but also on the level of transformation of the food items. Specifically, compared with Young Old and Old Old adults who named transformed food significantly better than natural food, Centenarians named natural food better than transformed food. This pattern of results was replicated in the word-picture matching task, suggesting that the degradation is at the representational level, and not at the output level. Consistently, the analysis of the naming errors revealed that Centenarians made more semantic errors with transformed food than with natural food, whereas Young Old adults and Old Old adults displayed the opposite pattern. In the next section we will discuss these results in turn.

First, experience affects food recognition: Centenarians reported to have consumed transformed food less frequently than natural food in their life, a pattern that was not observed in Young Old and Old Old adults. Results from the regression and bootstrap analyses corroborated the interpretation that the frequency of food consumption effectively predicted Centenarians’ naming accuracy. Experience of specific types of food may be considered as a category learning process that has been shown to affect object recognition^37^, even if such process took place in a large span of time, as in the case of our participants. Our results resemble those observed in the study by Poon and Fozard^27^ about obsolete and contemporary object naming in young and elderly adults. If experience is really at stake in our study, as the data strongly suggest, future centenarians should show a similar but inverse pattern of performance, being better than contemporary centenarians at recognizing transformed food.

Second, our study also shows that semantic memory declines later life, as observed in the naming task, and that this decline is particularly sharp in the 10^th^ decade of life. Interestingly, also different psycholinguistic variables such as age of acquisition, familiarity and word frequency of food items vary in their predictive value depending on the age of participants. For instance, in Centenarians, food items that are less familiar, less frequent and acquired later in life are more difficult to be recognized. This is consistent with the decline of episodic memory affecting first recent events while leaving long-term memories better preserved (for a review^38^). This pattern of degradation is consistent with the correlation analyses that revealed a significant positive correlation between natural food naming performance and familiarity, and an inverse correlation with age of acquisition, which was not observed for transformed food items. In addition, when we considered caloric content, found to predict food naming performance^34^, we observed that it positively correlated with naming transformed food in Young Old and Old Old adults, but not in Centenarians. The richer energetic content of transformed food seems to be highly salient for the Young Old and Old Old groups (see also^34^) and less so for Centenarians, possibly because these individuals need less energy (see for instance^39^), or because of their lower familiarity with this food category. In contrast, participants’ naming performance on natural food items, that are lower in calories than transformed foods, does not correlate with their caloric content. Thus our findings suggest that the distinction between natural and transformed food is biologically relevant, as suggested elsewhere^36,40–44^.

This study has some limitations. As healthy centenarians are rare, our sample size is inevitably small. Moreover, there is a gap in our sample as we excluded the participants with more than 91 years of age, while in other studies (see for instance^45^), people above 95 were considered as ‘centenarians’. As we aimed at testing group-specific effects, we avoided overlaps between the Old Old and the Centenarians groups. In addition, Young Old and Old Old adults perform at ceiling in the word-picture matching task, which limited us to draw conclusions only concerning centenarians. As a last point, due to the length of the battery and the fragility of the participants, in particular Centenarians, we limited the neuropsychological testing to the MMSE.

## Conclusion

Centenarians recognize more efficiently natural rather than transformed food, whereas the other two age groups show the opposite pattern, recognizing transformed food better than natural food. This result and the different life-long eating habits of our age-cohorts, strongly suggest that the lexical-semantic processes underlying object recognition are partly shaped by experience. This aspect deserves to be further explored, perhaps by investigating other relevant semantic categories that may yield differences in cohorts such as obsolete versus contemporary technological equipment or types of vehicles. Finally, our results also show that semantic memory, even though is quite resilient to the ageing process, eventually declines, with a sharper and more dramatic decline occurring in the 10^th^ decade of life.

## Materials and Methods

### Participants

The study enrolled 61 participants, belonging to three groups: 24 Young Old adults (15 F, age range 51–74), 19 Old Old adults (13 F, 75–91) and 18 Centenarians (12 F, 100–108). The inclusion criteria were: participants were not affected by neurodegenerative diseases, were native Italian speakers and had at least five years of education (except for three Centenarians, who did not reach 5 years of education). Participants were administered the MMSE^46^. A raw MMSE score >25 was accepted for Young Old and Old Old adults, while a MMSE score >20 was accepted for Centenarians. Three Centenarians had significantly lower MMSE scores, due to motor deficits and weakness that made it impossible for them to perform some tasks. However, they were screened by a neurologist and were diagnosed as cognitively normal according to the Criteria of the Diagnostic and Statistic Manual of Mental Disorders – IV^47^ (for the full screening protocol, see^48^). Moreover, participants were screened for deficits affecting vision and deafness. Participants signed an informed consent form and were tested in accordance to the relevant guidelines for experiments involving human subjects as stated by the declaration of Helsinki^49^. The study was approved by SISSA’s Ethics Committee.

### Experimental Tasks and Procedure

All participants (N = 61) were administered with the following experimental tasks in a fixed order: categorization, picture naming and word-picture matching. Tasks were presented on a computer using Microsoft PowerPoint presentations controlled by the experimenter. The experimenter recorded the participants’ responses on a dedicated scoring sheet. Before each task, participants performed an 8-trial practice in order to familiarize with the task.

### Experimental Stimuli

Seventy-two images representing both food and non-food items were used in the study, with 18 photographs of transformed foods, 18 of natural foods, 18 natural non-edible objects and 18 kitchen utensils. All the images were selected from FRIDa database^50^ (resolution: 530 × 530 pixels). The corresponding names were matched for number of letters and written frequency (see^34^). Age of acquisition and familiarity rates about these stimuli were derived from a norming study described in^34^.

### Task 1 – Categorization

This task investigates the ability to categorize both food (natural and transformed) and non-food items. The experiment contained 144 trials arranged in 4 blocks, with each trial consisting in two pictures. Each block contained 36 trials, with half of the trials consisting of two items belonging to the same category (N = 18, same trials), and the other half consisting of two items belonging to different categories (N = 18, different trials). Participants were asked to decide whether the two items were either two foods (natural or transformed) or two non-foods, or whether they were a food and a non-food.

### Task 2 – Confrontation Naming

This task taps the ability to retrieve name of the item depicted in the picture. The same 72 pictures of the categorization task were used. Each image was presented at the centre of the computer screen. Participants were asked to name the picture.

### Task 3 – Word -Picture matching

This task aims at evaluating the ability of an individual to comprehend nouns. On each trial, the experimenter uttered the name of an item and the participant was required to point to the target among six pictures of which five were distractors (a semantic distractor belonging to the same category as the target, and the remaining to the other categories). For instance, for natural food, in addition to the apricot as a target, and a mandarin as a semantic distractor, there were four more distractors: a knife, a leaf, a slice of pizza and a root. The six pictures were randomly placed in each of the six possible locations, three on the upper part of the screen and the other three on the lower part. A total of 72 trials were administered, with the target items being the same as in Tasks 1–2.

### Data analysis

Central tendency analyses were performed on the data for each of the tasks (Supplementary Information S1).

For the naming task, we performed a repeated-measures MANCOVA with Group (Young Old adults, Old Old adults, Centenarians) as between-subjects factor, Type of item (food, non-food) and Level of artificiality (natural versus artificial items) as within-subjects factors and Education level and MMSE score as covariates. To further investigate the interaction group x Type of item x Level of artificiality, we ran two MANCOVAs Group x Level of artificiality, separately for the food and non-food type of stimulus. Multiple comparisons were Bonferroni-corrected. For the categorization and the word- picture matching tasks we ran, due to the violation of normality of the data, Mann-Whitney tests to compare the overall performance of three groups and Wilcoxon rank sum tests to investigate the differences in the performance at food and non-food and natural and transformed food within each of the groups.

In addition, we also analysed the data using generalized linear mixed models with binomial family (see for instance^51^), with accuracy as dependent variable, *age group x stimulus type* as fixed factor, education as a covariate and *subject* as random factor for food vs. non-food. In order to explore the differences between natural and transformed food, we used a similar model, with age group x food type as fixed factor. Multiple comparisons were Bonferroni corrected and post-hoc analyses were performed with a Tukey test.

A series of regression and correlation analyses was calculated between naming of food and the psycholinguistic variables (frequency of use, word length, age of acquisition and familiarity) in each of the groups of participants in order to understand the role of each of these variables during aging. In addition, for food items only, naming performance was also correlated with the caloric content. These analyses focused on food only since food is the category for which, even thought we could not manipulate expertise, we could assess the level of experience with.

For what concerns the naming task, regarding food items, we also analyzed error types (anomias, semantic and verbal paraphasias, visual errors, good circumlocutions, poor circumlocutions) to investigate their lexical or semantic origin.

Finally, for both natural foods (raw fruit and vegetables) and transformed foods (pasta, sweets and cakes, processed meats), we asked Centenarians and a subset of our younger participants (aged 70–91) how ‘often’, ‘seldom’, or ‘never’ they consumed a particular item in their life. The aim was to investigate whether eating habits influence food recognition. Based on these data, a bivariate linear regression was carried out to investigate the quantitative relationship between naming performance and dietary habits and the bootstrap method^52^ was applied to the regression coefficient to estimate the confidence interval. All analyses were performed with statistical software PASW Statistics for Windows, Version 18.0. Chicago, SPSS Inc, except for the generalized linear mixed models, that were carried out using R (version 2.10.1; http://www.r-project.org/).

### Data availability

The datasets generated during and/or analysed during the current study are included in this published article (and its Supplementary Information files).

## Electronic supplementary material

Dataset4

Dataset6

Dataset1

Dataset2

Datset3

Dataset5

Supplementary Information
