# Supplementary material for: How experience modulates semantic memory for food: evidence from elderly adults and centenarians
Source: Sci Rep. 2018 Apr 24;8:6468. doi: 10.1038/s41598-018-24776-3 (PMC5915576; doi:10.1038/s41598-018-24776-3)
Supplement: Supplementary file 7 — Supplementary Information [file 41598_2018_24776_MOESM7_ESM.docx]

**Supplementary Information**

**How experience modulates semantic memory for food: evidence from elderly adults and centenarians**

Miriam Vignando* ^1^, Marilena Aiello ^1^, Francesco Foroni^2^ , Gabriella Marcon^3,4^, Mauro Tettamanti^5^, & Raffaella I. Rumiati^1,6^.

1.Neuroscience and Society Laboratory, SISSA, Trieste, Italy

2.School of Psychology, Faculty of Health Sciences, Australian Catholic University, Sydney, Australia

3.Dept. of Medical and Biological Sciences, University of Udine, Italy

4.Dept. of Medical, Surgical and Health Sciences, University of Trieste, Italy

5.Laboratory of Geriatric Neuropsychiatry, IRCSS - Istituto di Ricerche Farmacologiche Mario Negri, Milan, Italy

6.ANVUR, Italy

**Running title**: Semantic Memory for Food

**Corresponding author**:

Miriam Vignando: [mvignand@sissa.it](mailto:mvignand@sissa.it)

Via Bonomea 265

34136

Trieste (TS)

Italy

**S1.** Central tendency measures for each of the categories and each task (a) and across groups (b).

a)


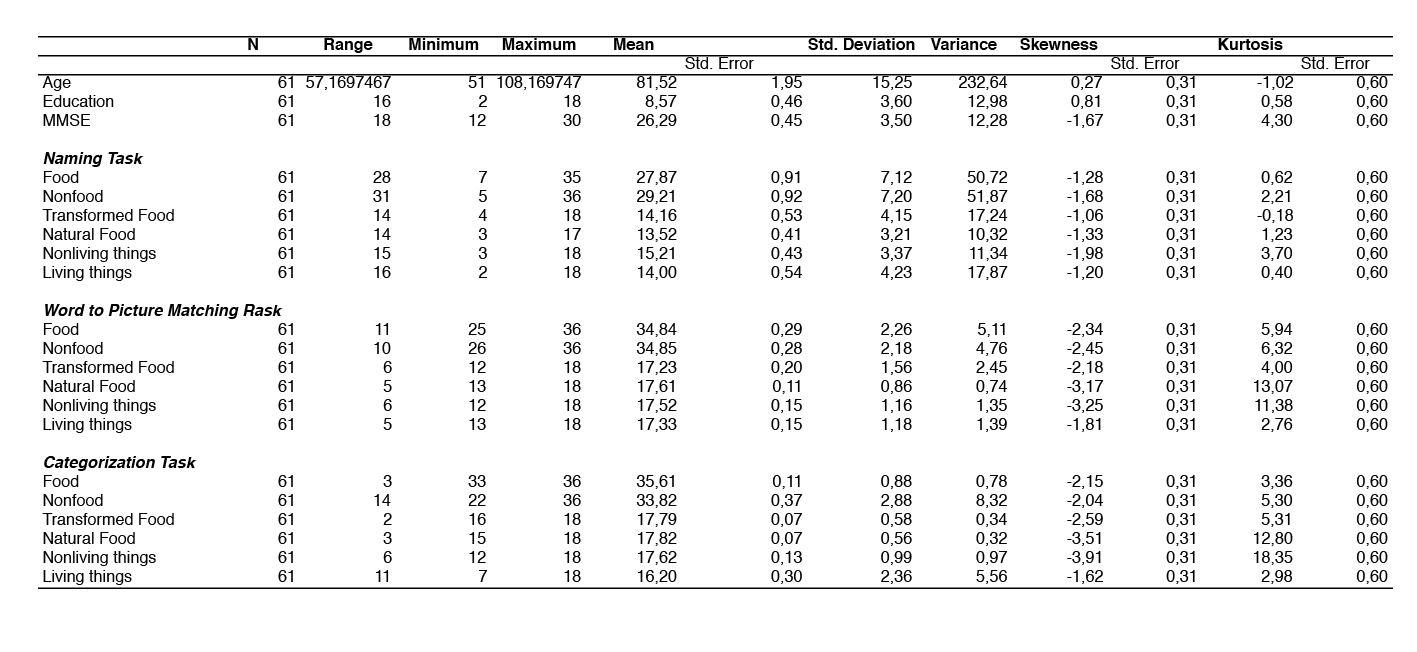


b)

|  | ***Naming*** |  |  |  |  |  |  |  |
| --- | --- | --- | --- | --- | --- | --- | --- | --- |
|  |  |  | Food | Nonfood | TF | NF | Nonliving | Living |
| ***Young Old Adults*** | N |  | 24 | 24 | 24 | 24 | 24 | 24 |
|  | Mean |  | 32.21 | 33.33 | 16.75 | 15.42 | 16.96 | 16.38 |
|  | S.E.M |  | 0.36 | 0.45 | 0.23 | 0.25 | 0.24 | 0.37 |
|  | Std. Dev. |  | 1.77 | 2.22 | 1.11 | 1.21 | 1.16 | 1.81 |
|  |  |  |  |  |  |  |  |  |
| ***Old Old Adults*** | N |  | 19 | 19 | 19 | 19 | 19 | 19 |
|  | Mean |  | 31.21 | 32.37 | 16.16 | 14.63 | 16.32 | 16.05 |
|  | S.E.M |  | 0.64 | 0.48 | 0.45 | 0.37 | 0.27 | 0.39 |
|  | Std. Dev. |  | 2.78 | 2.09 | 1.98 | 1.61 | 1.16 | 1.72 |
|  |  |  |  |  |  |  |  |  |
| ***Centenarians*** | N |  | 18 | 18 | 18 | 18 | 18 | 18 |
|  | Mean |  | 18.56 | 20.39 | 8.61 | 9.83 | 11.72 | 8.67 |
|  | S.E.M |  | 1.41 | 1.74 | 0.69 | 0.78 | 1.01 | 0.84 |
|  | Std. Dev. |  | 5.97 | 7.37 | 2.91 | 3.29 | 4.27 | 3.56 |
|  |  |  |  |  |  |  |  |  |
|  | ***Word-Picture Matching*** | |  |  |  |  |  |  |
|  |  |  | Food | Nonfood | TF | NF | Nonliving | Living |
| ***Young Old Adults*** | N |  | 24 | 24 | 24 | 24 | 24 | 24 |
|  | Mean |  | 36.00 | 35.96 | 18.00 | 18.00 | 18.00 | 17.96 |
|  | S.E.M |  | 0.00 | 0.04 | 0.00 | 0.00 | 0.00 | 0.04 |
|  | Std. Dev. |  | 0.00 | 0.20 | 0.00 | 0.00 | 0.00 | 0.20 |
|  |  |  |  |  |  |  |  |  |
| ***Old Old Adults*** | N |  | 19 | 19 | 19 | 19 | 19 | 19 |
|  | Mean |  | 35.84 | 35.79 | 17.89 | 17.95 | 17.89 | 17.89 |
|  | S.E.M |  | 0.12 | 0.10 | 0.11 | 0.05 | 0.07 | 0.07 |
|  | Std. Dev. |  | 0.50 | 0.42 | 0.46 | 0.23 | 0.32 | 0.32 |
|  |  |  |  |  |  |  |  |  |
| ***Centenarians*** | N |  | 18 | 18 | 18 | 18 | 18 | 18 |
|  | Mean |  | 32.22 | 32.39 | 15.50 | 16.72 | 16.50 | 15.89 |
|  | S.E.M |  | 0.64 | 0.64 | 0.47 | 0.28 | 0.41 | 0.30 |
|  | Std. Dev. |  | 2.73 | 2.73 | 1.98 | 1.18 | 1.76 | 1.28 |
|  |  |  |  |  |  |  |  |  |
|  | ***Categorization*** | |  |  |  |  |  |  |
|  |  |  | Food | Nonfood | TF | NF | Nonliving | Living |
| ***Young Old Adults*** | N |  | 24 | 24 | 24 | 24 | 24 | 24 |
|  | Mean |  | 35.92 | 32.54 | 18.000 | 17.917 | 17.375 | 15.167 |
|  | S.E.M |  | 0.08 | 0.75 | 0.000 | 0.083 | 0.275 | 0.589 |
|  | Std. Dev. |  | 0.41 | 3.68 | 0.000 | 0.408 | 1.345 | 2.884 |
|  |  |  |  |  |  |  |  |  |
| ***Old Old Adults*** | N |  | 19 | 19 | 19 | 19 | 19 | 19 |
|  | Mean |  | 35.84 | 35.00 | 17.84 | 18.00 | 17.89 | 17.11 |
|  | S.E.M |  | 0.12 | 0.37 | 0.12 | 0.00 | 0.11 | 0.30 |
|  | Std. Dev. |  | 0.50 | 1.63 | 0.50 | 0.00 | 0.46 | 1.33 |
|  |  |  |  |  |  |  |  |  |
| ***Centenarians*** | N |  | 18 | 18 | 18 | 18 | 18 | 18 |
|  | Mean |  | 34.94 | 34.28 | 17.56 | 17.39 | 17.67 | 16.61 |
|  | S.E.M |  | 0.30 | 0.48 | 0.18 | 0.22 | 0.18 | 0.47 |
|  | Std. Dev. |  | 1.26 | 2.02 | 0.78 | 0.92 | 0.77 | 1.97 |
|  |  |  |  |  |  |  |  |  |
|  |  |  |  |  |  |  |  |  |

*As shown by the skewness and kurtosis values reported in the table above, we ran parametric tests for the naming task, whereas nonparametric statistics for the Word to picture matching and the Categorization tasks were carried out. This lack of normality in these two tasks is due to the ceiling effect reached by controls in the Word to picture matching task and by all of our groups in the Categorization task.*

**S2.** Mann Whitney and Wilcoxon rank sum tests results for non-food at the Word to Picture Matching task.

a)


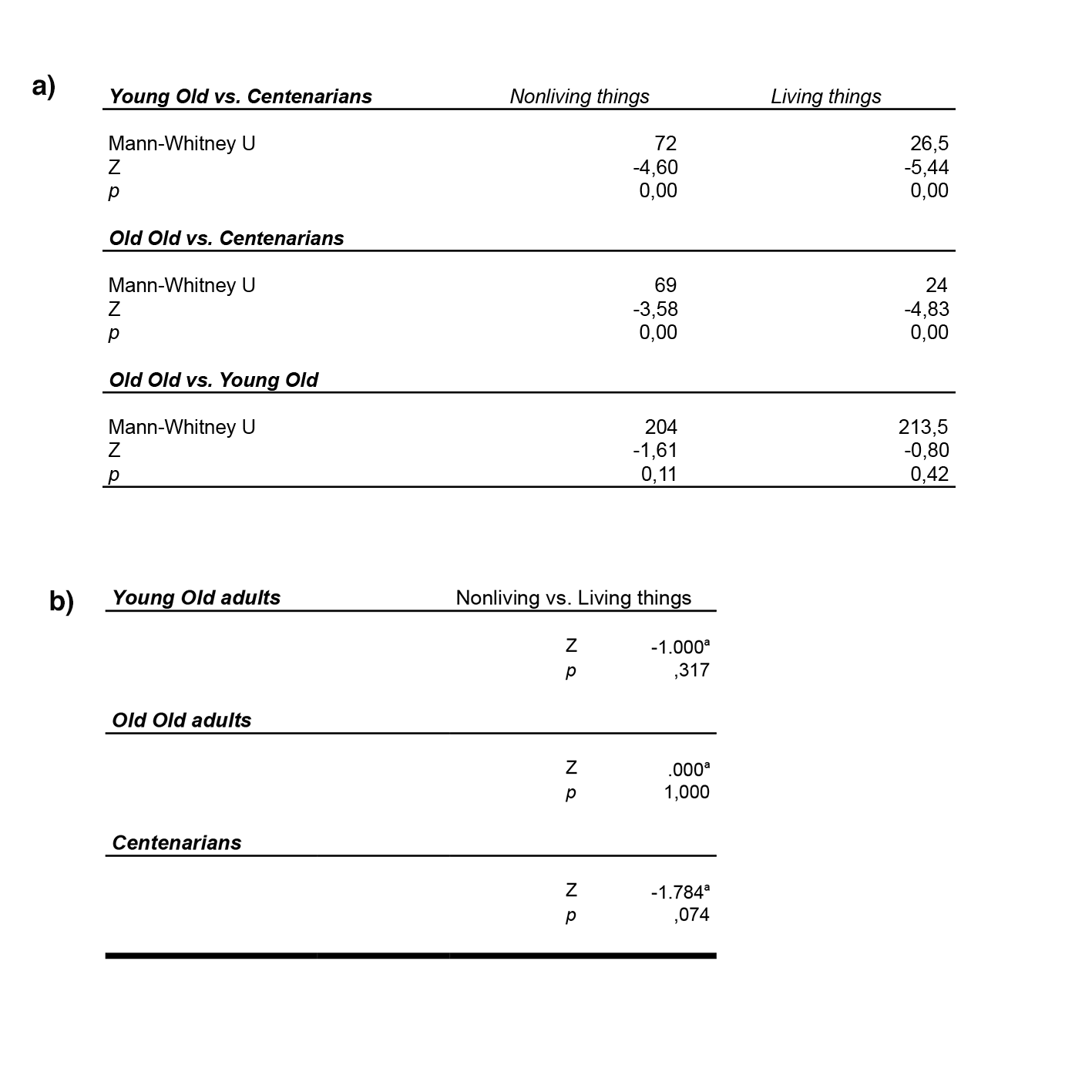


b)


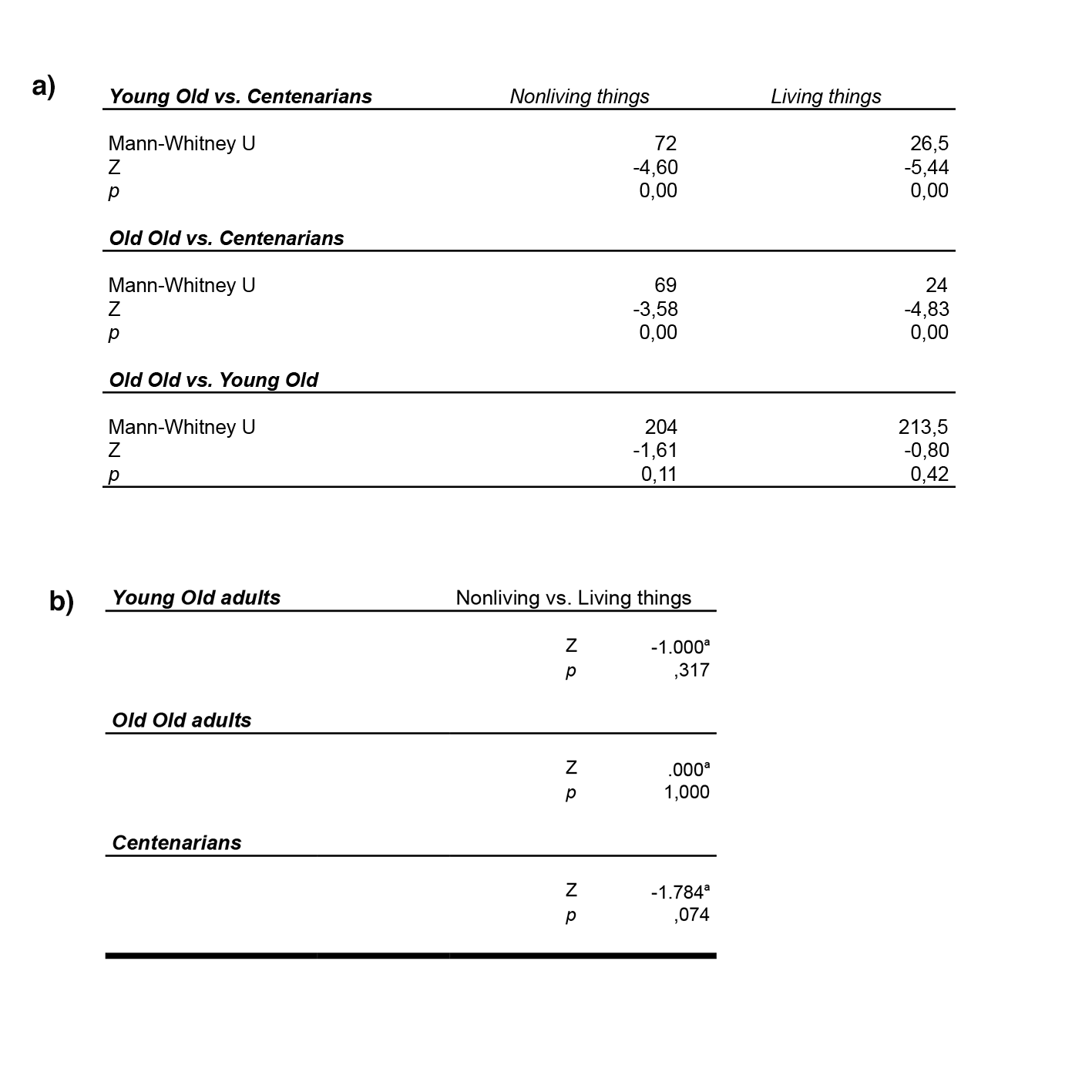


*a) A significant difference in performance is observed between Young Old adults and Centenarians and Old Old adults and Centenarians, whereas Young Old and Old Old adults show no significant differences in performance. b) No significant differences were observed between recognition of living things and nonliving things in none of the age groups (see S1 for Young Old and Old Old, performing at ceiling) as observed with the Wilcoxon rank sum test performed only for Centenarians.*

**S3**. Mean and ranking of error types performed overall by Centenarians.


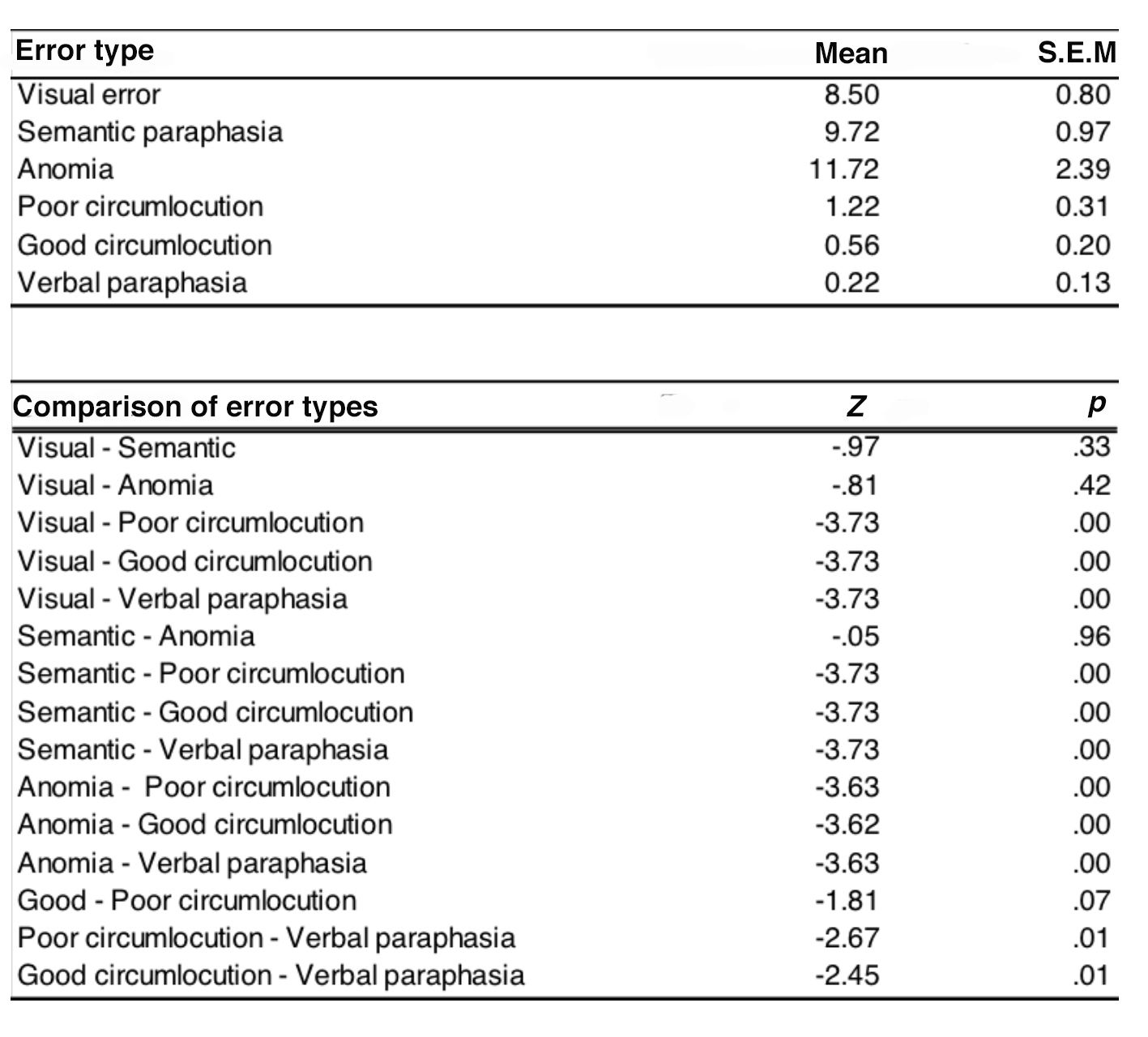


*As mentioned in the manuscript, the most frequent error types performed by Centenarians are visual errors, semantic errors and anomias. No significant difference has emerged between the amount of each of these three error types. However, visual errors, semantic paraphasias and anomia are significantly more frequent than any other error type considered in the analysis.*

**S4**. Differences in error types between natural and transformed food in Centenarians, Old Old adults and Young Old adults.

*As reported in the manuscript, Centenarians do more semantic errors for transformed food vs. natural food, whereas both Young Old and Old Old adults show the opposite pattern.*

**S5**. Pearson correlations on psycholinguistic variables and food naming performance

*Centenarians name food items better if they have a high written frequency, are highly familiar and were acquired earlier in life. Old Old adults’ performance is affected only by familiarity, whereas none of the psycholinguistic variables correlates with Young Old adults’ naming performance.*

**S6.** Regression analyses on psycholinguistic variables and food naming performance


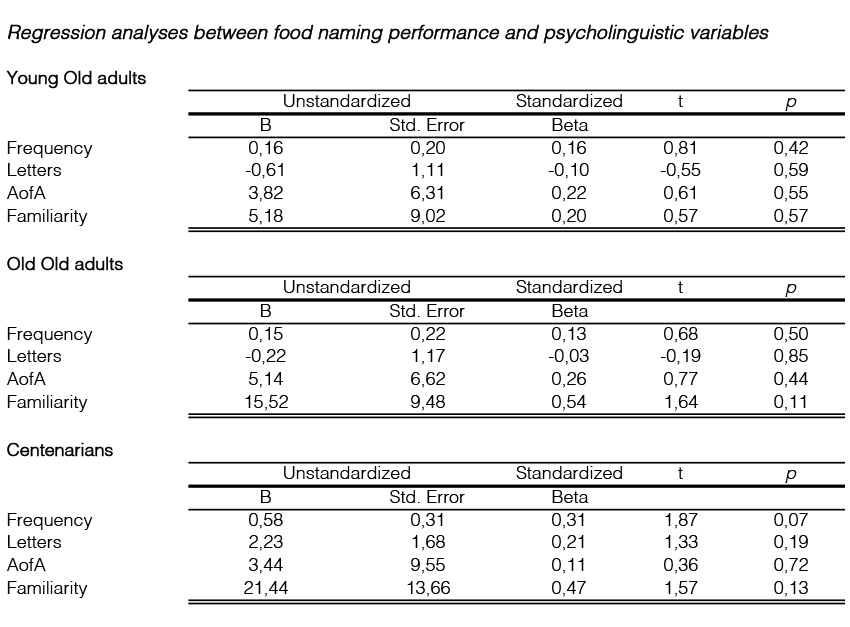


**S7.** Regression analyses on natural and transformed food naming performance and age of acquisition and familiarity.

**S8.** Mann-Whitney U tests and Wilcoxon rank sum tests for the Categorization task.

**
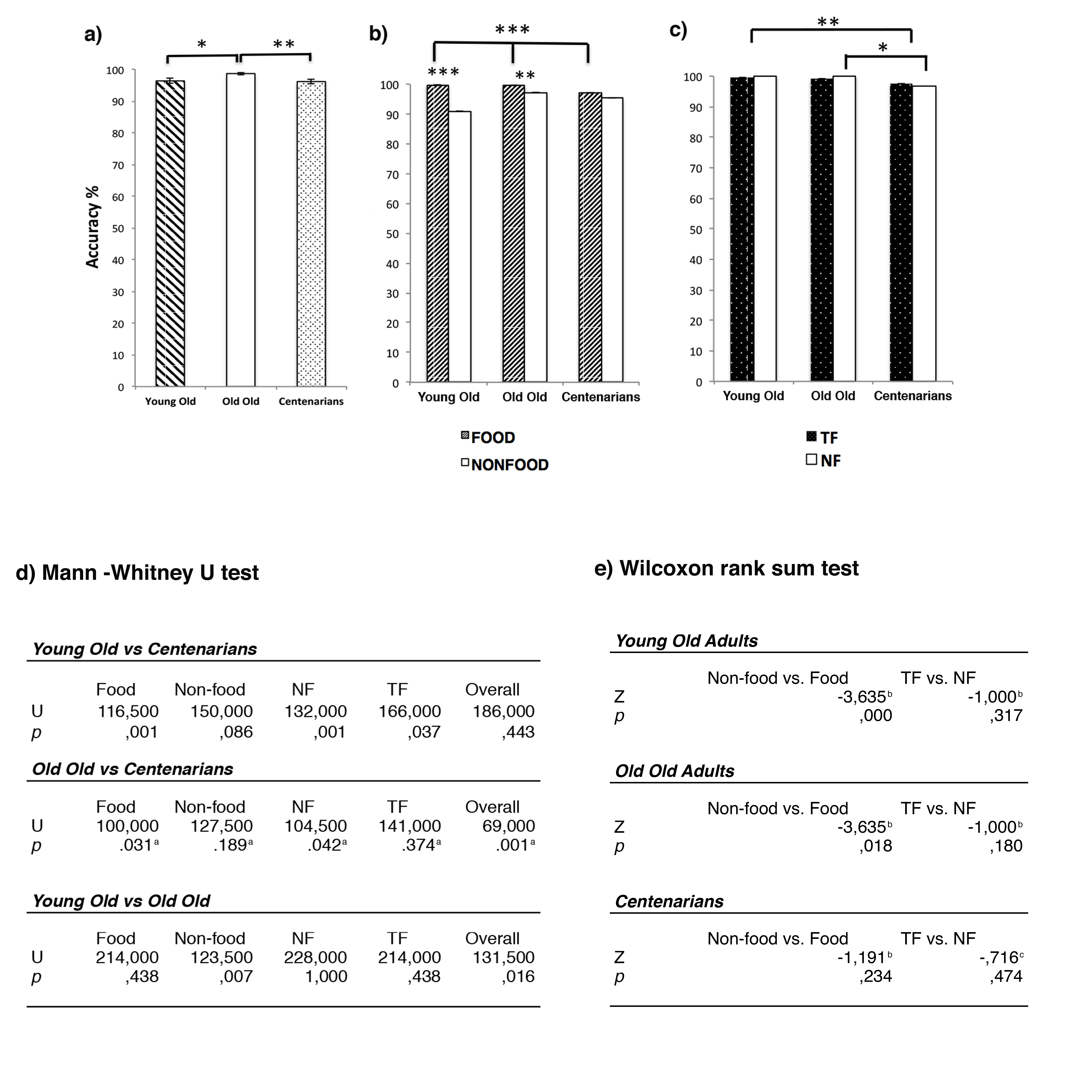
**

*a) The Mann-Whitney U test revealed, overall, a significant difference was observed only between categorization accuracy of Centenarians and Old Old Adults, but not Young Old adults, with Centenarians performing significantly worse. Moreover, Old Old adults perform significantly better than Young Old adults as well. However, as shown by the histogram, all groups perform at ceiling (> 95% accuracy).*

*b) Concerning food only, Mann-Whitney U tests showed that both Young Old and Old Old adults perform significantly better than centenarians; concerning non-food, Old Old adults are significantly more accurate than Young Old adults. Moreover, Wilcoxon rank sum tests revealed that both Young Old and Old Old adults categorize food significantly better than nonfood.*

*c) Young Old adults perform better than Centenarians at both natural and transformed food categorization, whereas Old Old adults show a better performance for natural food only. No differences have been observed between Young Old and Old Old adults. Wilcoxon rank sum tests revealed no significant differences between categorization of these two categories for none of the three groups.*

*d) Mann-Whitney U tests and e) Wilcoxon rank sum tests statistics.*

S9. Results for each of the three tasks analysed with linear mixed models with binomial family, with R programming.

1. *As shown in the table below, food there is a significant effect of ‘group’, with Centenarians performing significantly worse than Young Old and Old Old adults. No differences between food and non-food are observed. Education was entered as a covariate, with no significant effect. MMSE was entered as a covariate as well, given the significantly worse performance of Centenarians, but no significant effect was observed.*

| ***Naming: Food vs. Nonfood*** | |  |  |  |  |
| --- | --- | --- | --- | --- | --- |
| Generalized linear mixed model fit by maximum likelihood (Laplace Approximation) ['glmerMod'] | | | | | |
| Family: binomial ( logit ) | |  |  |  |  |
| Formula: Accuracy ~ Age group * Stimulus (Food vs. Nonfood) + Education + MMSE + (1 \| ID) | | | | | |
| Control: glmerControl (optimizer = "bobyqa", optCtrl = list (maxfun = 1e+05)) | | | | |  |
|  |  |  |  |  |  |
| Random effects: |  |  |  |  |  |
|  | Variance | Std.dev |  |  |  |
| ID (Intercept) | 0.29 | 0.54 |  |  |  |
| Number of obs: 4392, groups: ID, 61 | |  |  |  |  |
|  |  |  |  |  |  |
| **Fixed effects:** |  |  |  |  |  |
|  |  | **Estimate** | **Std.error** | **z** | **Pr(>\|z\|)** |
| (intercept) |  | 0.15 | 0.69 | 0.22 | 0.08 |
| Old Old vs. Centenarians | | 1.97 | 0.27 | 7.16 | 7.91e-13*** |
| Young Old vs. Centenarians | | 2.29 | 0.26 | 8.92 | <2e-16*** |
| Stimulus (food vs. nonfood) | | -0.17 | 0.12 | -1.46 | 0.14 |
| Education |  | -0.02 | 0.02 | -0.93 | 0.35 |
| MMSE |  | 0.01 | 0.03 | 0.48 | 0.63 |
| Old Old * Stimulus | | -0.04 | 0.21 | -0.2 | 0.84 |
| Young Old * Stimulus | | 0.09 | 0.21 | 0.41 | 0.68 |
|  |  |  |  |  |  |
|  |  |  |  |  |  |
| ***Naming Natural vs. Transformed Food*** | |  |  |  |  |
| Formula: Accuracy ~ Age group * Stimulus_type (NF vs TF) + Calorie Content + Education + MMSE + (1 \| ID) | | | | | |
|  |  |  |  |  |  |
| Random effects: |  |  |  |  |  |
|  | Variance | Std.dev |  |  |  |
| ID (Intercept) | 0.3 | 0.54 |  |  |  |
| Number of obs: 2196, groups: ID, 61 | |  |  |  |  |
|  |  |  |  |  |  |
| Fixed effects: |  |  |  |  |  |
|  |  | **Estimate** | **Std. Error** | **z value** | **Pr(>\|z\|)** |
| (intercept) |  | -0.51 | 0.81 | -0.63 | 0.53 |
| Old Old Adults vs. Centenarians | | 2.45 | 0.32 | 7.53 | 4.93e-14 *** |
| Young Old Adults vs. Centenarians | | 3 | 0.31 | 0.12 | < 2e-16 *** |
| Foodtype |  | 1 | 0.28 | 3.01 | 9.14e-05 *** |
| Education |  | -0.04 | 0.027 | -1.32 | 0.18 |
| Calorie content |  | 1.10 | 0.28 | 3.91 | 0.0001*** |
| MMSE |  | -0.01 | 0.03 | -0.30 | 0.77 |
| Old Old adults*Foodtype | | -0.65 | 0.29 | -2.23 | 0.03* |
| Young Old adults*Foodtype | | -0.64 | 0.3 | -2.10 | 0.04* |
|  |  |  |  |  |  |
| Post hoc: Tukey test | |  |  |  |  |
|  |  |  |  |  |  |
| Contrast |  | **Estimate** | **Std. Error** | **z value** | **p** |
| Centenarians: NF vs. TF | | -1.1 | 0.28 | -3.91 | 0.001 |
| Old Old: NF vs. TF | | -0.45 | 0.32 | -1.42 | 0.72 |
| Young Old: NF vs. TF | | -0.46 | 0.33 | -1.42 | 0.72 |
|  |  |  |  |  |  |
| Results are given on the log odds ratio (not the response) scale. | | | |  |  |
| P value adjustment: tukey method for comparing a family of 6 estimates | | | | |  |
| Signif. codes: 0 ‘***’ 0.001 ‘**’ 0.01 ‘*’ 0.05 ‘.’ 0.1 ‘ ’ 1 | | |  |  |  |

*For naming natural versus transformed food there is a significant effect of ‘group’, with Centenarians performing significantly worse than Young Old and Old Old adults and significant interactions between Young Old and Old Old performance and foodtype, relative to Centenarians. Post-hoc tests (Tukey) revealed that, while Young Old and Old Old do not perform significantly better at any of the categories of interest, Centenarians perform significantly worse at transformed, rather than natural food. Education was initially entered as a covariate but was subsequently removed since it resulted as not significant.*

*b) Results for the word-picture matching task are obtained only for Old Old versus Centenarians, due to the lack of variance and to the ceiling effect observed in the Young Old group. As observed with the naming task, there is no difference at food and non-food recognition in any of the groups. Education was entered as a covariate, with no significant effect. MMSE was entered as a covariate as well, given the significantly worse performance of Centenarians, but no significant effect was observed. For what concerns natural and transformed food, a main effect of foodtype is present, with both groups recognizing natural food better.*

| ***Word-picture matching: Food vs. Nonfood*** | | |  |  |  |
| --- | --- | --- | --- | --- | --- |
| Formula: Accuracy ~ Age group * Stimulus (Food vs. Nonfood) + Education + MMSE+ (1 \| ID) | | | | | |
|  |  |  |  |  |  |
| Random effects: |  |  |  |  |  |
|  | Variance | Std.dev |  |  |  |
| ID (Intercept) | 0.37 | 0.6 |  |  |  |
| Number of obs: 2664, groups: subject, 37 | |  |  |  |  |
|  |  |  |  |  |  |
| **Fixed effects:** |  |  |  |  |  |
|  |  | **Estimate** | **Std. Error** | **z value** | **Pr(>\|z\|)** |
| (intercept) |  | -1.58 | 1.03 | -1.54 | 0.13 |
| Old Old vs. Centenarians | | 2.79 | 0.59 | 4.72 | 2.39e-06 *** |
| Stimulus (food vs. nonfood) | | -0.04 | 0.84 | -0.47 | 0.64 |
| Education |  | -0.03 | 0.05 | -0.56 | 0.58 |
| MMSE |  | 0.06 | 0.04 | 1.39 | 0.16 |
| Old Old * stimulus |  | 0.34 | 0.78 | 0.44 | 0.66 |
|  |  |  |  |  |  |
|  |  |  |  |  |  |
|  |  |  |  |  |  |
| ***Word-picture matching: Natural vs. Transformed food*** | | | |  |  |
| Formula: Accuracy ~ Age group * Stimulus_type (NF vs TF) + Education + MMSE+ (1 \| ID) | | | | | |
|  |  |  |  |  |  |
| Random effects: |  |  |  |  |  |
|  | Variance | Std.dev |  |  |  |
| ID (Intercept) | 0.38 | 0.62 |  |  |  |
| Number of obs: 1332, groups: subject, 37 | |  |  |  |  |
|  |  |  |  |  |  |
| **Fixed effects:** |  |  |  |  |  |
|  |  | **Estimate** | **Std. Error** | **z value** | **Pr(>\|z\|)** |
| (intercept) |  | 0.49 | 1.16 | 0.43 | 0.67 |
| Old Old vs. Centenarias |  | 3.06 | 0.79 | 3.87 | 0.000109*** |
| Foodtype (NF) |  | 0.77 | 0.27 | 2.86 | 0.004** |
| Education |  | 0.04 | 0.06 | 0.73 | 0.5 |
| MMSE |  | 0.05 | 0.47 | 1.09 | 0.28 |
| Old Old * stimuls type | | -0.08 | 1.25 | -0.06 | 0.952 |
|  |  |  |  |  |  |

*c) Results for the categorization task. Education was entered as a covariate, not showing any significant effect. Given the fact that at this task all groups perform at ceiling, MMSE scores were not used as covariates. Results show a main effect of group, with Old Old performing significantly better than Centenarians. Moreover, there is a main effect of the stimulus, with food being better categorized than non-food and a significant interaction between group and stimulus. Post-hoc tests revealed that Old Old and Young Old adults, but not Centenarians, categorize food better with respect to non-food.*

| ***Categorization: Food vs. Nonfood*** | |  |  |  |  |
| --- | --- | --- | --- | --- | --- |
| Formula: Accuracy ~ Age group * Stimulus (Food vs. Nonfood) + Education + (1 \| ID) | | | | | |
|  |  |  |  |  |  |
|  |  |  |  |  |  |
| Random effects: |  |  |  |  |  |
|  | Variance | Std.dev |  |  |  |
| ID (Intercept) | 1.07 | 1.03 |  |  |  |
| Number of obs: 8784, groups: ID, 61 | |  |  |  |  |
|  |  |  |  |  |  |
| **Fixed effects:** |  |  |  |  |  |
|  |  | **Estimate** | **Std. Error** | **z value** | **Pr(>\|z\|)** |
| (intercept) |  | 3.16 | 0.28 | 11.25 | < 2e-16 *** |
| Old Old |  | 1.22 | 0.43 | 2.82 | 0.005** |
| Young Old |  | 0.05 | 0.37 | 0.12 | 0.9 |
| Stimulus (food vs. nonfood) | | 0.5 | 0.20 | 2.56 | 0.01* |
| Education |  | 0.01 | 0.01 | 0.32 | 0.75 |
| Old Old * stimulus |  | 0.91 | 0.45 | 2.02 | 0.04* |
| Young Old * stimulus | | 1.23 | 0.31 | 3.39 | 8.03e-05 *** |
|  |  |  |  |  |  |
| Post hoc: Tukey |  |  |  |  |  |
| Contrast |  | **Estimate** | **Std. Error** | **z value** | **p** |
| Centenarians: Food vs. Nonfood | | -0.5 | 0.20 | -2.56 | 0.1 |
| Old Old: Food vs. Nonfood | | -1.42 | 0.41 | -3.47 | 0.007 |
| Young Old: Food vs. Nonfood | | -1.73 | 0.24 | -7.13 | < .0001 |
|  |  |  |  |  |  |
| ***Categorization: Natural vs. Transformed Food*** | | |  |  |  |
| Formula: Accuracy ~ Age group * Stimulus_type (NF vs TF) + Calorie Content + (1 \| ID) | | | | | |
|  |  |  |  |  |  |
| Random effects: |  |  |  |  |  |
|  | Variance | Std.dev |  |  |  |
| ID (Intercept) | 1.62 | 1.27 |  |  |  |
| Number of obs: 2196, groups: ID, 61 | |  |  |  |  |
|  |  |  |  |  |  |
| Fixed effects: |  |  |  |  |  |
| Estimate Std. Error z value Pr(>\|z\|) |  | **Estimate** | **Std. Error** | **z value** | **Pr(>\|z\|)** |
| (intercept) |  | 3.57 | 0.52 | 6.9 | 5.17e-12 *** |
| Old Old |  | 18.47 | 43.58 | 0.42 | 0.67 |
| Young Old |  | 18.43 | 29.46 | 0.62 | 0.53 |
| Foodtype |  | 0.67 | 0.45 | 1.49 | 0.14 |
| Education |  | 0.16 | 0.12 | 1.31 | 0.19 |
| Old Old * Foodtype | | -17.24 | 43.6 | -0.39 | 0.69 |
| Young Old*Foodtype | | -16.53 | 29.45 | -0.56 | 0.56 |
| --- |  |  |  |  |  |
| Signif. codes: 0 ‘***’ 0.001 ‘**’ 0.01 ‘*’ 0.05 ‘.’ 0.1 ‘ ’ 1 | | |  |  |  |
